# Workout at a virtual gym: Surrounding avatar’s motion speed and exercise intensity effect on the user’s time perception

**DOI:** 10.1371/journal.pone.0311860

**Published:** 2024-12-06

**Authors:** Bingcheng Ke, Tzu-Yang Wang, Takaya Yuizono, Hideaki Kanai

**Affiliations:** Graduate School of Advanced Science and Technology, Japan Advance Institute of Science and Technology, Nomi, Ishikawa, Japan; The University of Edinburgh, UNITED KINGDOM OF GREAT BRITAIN AND NORTHERN IRELAND

## Abstract

This study explored the relationship between surrounding avatars and time perception in a virtual reality (VR) gymnasium. Previous research has highlighted that motion speed and exercise intensity significantly influence time perception. In VR, time perception is shaped by various factors, such as an avatar’s embodiment at different levels. However, the specific effects of the surrounding avatar on time perception in a VR gymnasium context remain unclear. Thus, this study focuses on two key attributes of the surrounding avatar: (1) motion speeds and (2) exercise intensity. Participants in a VR gymnasium either rode a stationary bike or sat on one while observing avatars performing exercises in the virtual environment(VE). They were then asked to estimate the duration judgment and the feeling of the passage of time for each task. The results revealed that when the surrounding avatars exercised at a faster motion speed, participants perceived the duration of time as longer and felt that time passed more quickly. Additionally, high-intensity exercise led participants to perceive the passage of time as faster.

## Introduction

The rapid advancement of virtual reality (VR) technology has shown tremendous potential in various fields, particularly fitness and exercise. VR creates immersive computer-generated three-dimensional environments that make users feel as though they are in a virtual world [1]. This technology allows users to transit between diverse environments, such as beaches or tropical rainforests, and customizes environmental parameters to achieve an optimal environment for their needs. Consequently, an increasing number of individuals are gravitating towards VR software to replace conventional physical scenarios in daily life, owing to its manifold benefits. This study explored a VR gymnasium. This allows users to easily slip into their ideal workout atmosphere, alleviating boredom and loneliness [2].

An ideal VR gymnasium should enhance the users’ willingness to continue exercising. However, fatigue is a major threat that causes people to give up exercising [3]. Intense exercise in a gymnasium can lead to hyperarousal, causing people to perceive that time passes more slowly [4]., This perception of time dilation might decrease motivation, as participants feel that the activity takes longer and is more time-consuming [5]. Therefore, understanding how individuals perceive time in VR gyms is important when designing an ideal VR gymnasium.

### Time perception research in real world

Time perception refers to how people experience and understand time. It is subjective and can be affected by various factors in the physical world [6]. These factors are typically categorized as either external (e.g., environmental conditions) or internal (e.g., individuals’ physiological and psychological states).

External factors refer to various elements arising from the surrounding environment that influence time perception, such as the environment’s high heat acclimation, which can impair time awareness, and contribute to perceiving time longer [7]. Similarly, medicine that increases dopamine levels can prolong subjective time perception by affecting brains, for instance, high-concentration attention leads the brain to deal with extra information [8]. Environmental stimuli, such as music tempo and sound intensity also influence time perception. Specifically, slower tempos and louder sounds often lead to a distorted sense of time, making it feel longer [9, 10]. Visual stimulation, such as flicker rates, can also alter time perception and increase time estimates [11].

Internal factors, however, are linked to an individual’s inherent physiological and cognitive state on time perception. These factors can affect subjective time perception. For instance, conditions such as depression can slow time perception due to changes in brain function and neuroscience. People with mental illnesses generally experience time delay [12]. Emotions, such as fear and anxiety, can also impact time perception. Increased anxiety tends to make time feel longer because the heightened arousal from negative stimuli intensifies the perception of time passing slowly [13, 14]. Sex differences also played a role. Women typically perceive time intervals as being longer than men [15].

The internal clock model is widely accepted in psychological time research [16]. This model suggests that an internal clock system exists inside the human brain, including a pacemaker emitting pulses captured and accumulated by a switch, and the accumulated count can be used to judge and represent time intervals, that model could help individuals perceive time [17]. The model has been instrumental in understanding how various factors influence time perception, particularly in scenarios where time is distorted, such as during states of boredom when time seems to pass more slowly owing to reduced brain activity [2].

### Time perception research in VR

VR has enormous potential to offer immersive experiences that significantly affect time perception. Previous research has shown that while engaged in VR activities such as games or immersive videos, users often lose track of time, experiencing what is commonly called “time flying.” This effect is likely owing to the high level of engagement in VR, which distracts users from external time cues and body rhythms [18]. Schneider et al. attempted to use VR to relieve breast cancer pain during chemotherapy. They found that the patients’ duration judgment during VR-immersive chemotherapy underestimated the actual time by 28 percent, demonstrating how VR can distract attention from discomfort [19]. Lugrin et al. highlighted that comparing waiting times in the real world and virtual environment(VE) in the same situations, participants perceived time as being shorter than in real waiting room conditions because people focus more on the VR content, and the higher arousal, distracted their attention [20].

Recently, avatars have become the main target in VR time perception research. Unruh et al. highlighted that the presence of avatars and embodiments in VR can affect time perception. Participants reported the perceived time to be faster when they had self-controlled avatars [21]. Unruh et al. also investigated the effect of avatars with different levels of embodied representation, such as hands, arms, and the full body, which are controlled by the participants, on their time perception. They found that when participants controlled the avatar only with hands without any other part of the body, it contributed to the perception of time passing more slowly than in other conditions [22]. In a study by Landau et al., participants who embodied avatars with a child’s appearance perceived the duration of time in the VE as longer than those with other avatar appearances [23].

In addition to avatars, various virtual objects, and elements, such as audio-visual stimuli and object motion, influence time perception in VR. Picard et al. examined the effects of audio, visual, and combined stimuli when participants performed object-sorting tasks in a VE. Their results demonstrated that a combination of visual and audio stimuli significantly decreased the judgment of time duration. Furthermore, synchronized stimuli, such as virtual and audio elements, can lead to time compression or dilation, depending on the tempo [24]. Yamamoto and Miura explored the effect of the coherence of visual object motion on time perception. They found that participants who observed four line segments forming a coherent motion experienced altered time perception, which was influenced by perceived motion speed [25].

### Time perception research on exercise intensity

The relationship between exercise and time perception has been the focus of numerous studies, aimed at understanding the underlying mechanisms. For instance, Lambourne’s study on cycling found that participants perceived time to be approximately 15% during exercise than during rest. This can be explained by the internal clock model: cycling alters the internal clock pulse rate, leading to an overestimation of time [4]. Petrizzo et al. demonstrated that running can cause individuals to overestimate the time spent on visual stimulation tasks, suggesting that exercise acts as a distraction that influences time evaluation [26].

Exercise intensity is a vital factor affecting time perception during exercise. Hanson and Lee found that increased exercise intensity leads to slower perceived passage of time when people running at higher exertion levels like Rating of Perceived Exertion (RPE) of 17, and the estimated time is significantly lower than at moderate levels, such as RPE 13 [27]. Edwards and McCormick also found that exercise intensity distorted the participants’ time perception. The study found that performing Wingate cycles and rowing ergometer boats at slight and medium intensities did not affect time perception while exercising at maximum intensity caused participants to perceive a shorter duration [28]. Additionally, Hanson and Buckworth found that women chose to run at a relatively higher intensity when they could be selected by themselves, compared to men. Simultaneously, a higher intensity leads to a relatively slow feeling of time passage [29]. Moore and Olson indicated that time perception differed before and after high-intensity exercise. They tested people before and after exhaustive cycling and found that high-intensity exercise usually induced time distortion owing to fatigue. Under the fatigue state of people, duration judgment increases, and the relative error significantly increases [30].

In general, heightened sensory awareness and physical discomfort during intense exercise contribute to a longer perceived duration. This phenomenon is often linked to the release of catecholamines, which trigger hyperarousal—a state where the brain processes more information than usual, making time seem to pass more slowly than it does [31, 32].

However, while exercise intensity has been proven from people’s internal perspective will affect their time perception. But, the stimuli of exercise intensity from outside will also work on people’s time perception still not known, like the surrounding avatars. Thus, this is still important to investigate whether the intensity of the surrounding avatars affects people’s time perception.

### Time perception research on motion speed

The motion speed also significantly influenced time perception. For example, running compared with the static mode, running led people to percieve that time was going longer [33]. By contrast, Rietzler et al. found that controlling an avatar with a slowed-down motion in a VE results in a shorter estimated duration [34].

In addition to self-motion, external motion stimuli also affect time perception as well. Kaneko et al. demonstrated that participants in a virtual car perceive a longer time when experiencing a faster motion speed [35]. Similarly, Landeck et al. immersed participants in virtual tunnels with numerous different sections and found that a stronger sensation of self-motion and fast speed increased subjective time [36]. Additionally, Landeck’s further research showed that external object motion within VE, such as pendulum movements, caused time to be perceived as passing more quickly than simpler motions like linear or rotary because the complexity of the pendulum motion captured more of the participants’ attention [37]. Dynamic images and speed cues can influence people’s time perception, often causing them to overestimate the duration [38]. Another study compared a virtual object’s motion speed to a human movement speed, such as point-light displays (PLDs), with only a few points of light representing the major joints or key parts of a moving body. After watching themselves and other people’s dancing movements, which are recorded as PLDs at different speeds. The result showed that the PLDs’ moving speed also affects people’s time perception, with a faster motion speed of human movement frames leading to longer duration judgment [39].

In summary, both the internal and external motion speeds affect time perception. Images and PLDs with human motion information and speed properties are effective. In VR environments, such as VR gyms, the influence of motion speed is further compounded by social and interpersonal factors, where avatar behavior can introduce additional stimuli that affect users’ time perception [40]. Furthermore, human information, such as dynamic facial expressions and high-arousal emotional displays, induces a longer perceived duration [41]. Thus, displaying only PLDs, despite being understood as a human performance action, loses considerable information compared to avatars [42].

Hence, it is crucial to extend prior research on self-perceived motion speed and visual stimuli such as PLDs by investigating the impact of the surrounding avatar motion speed on individuals’ perception of time.

### Contributions

Previous research on avatars and time perception in VEs has focused on user-controlled avatars. However, the effect of surrounding avatars in VR gyms remains under-explored. The surrounding avatars, which can act as non-player characters (NPCs) or other users’ avatars, play a significant role in the social spaces of VR gyms. They differ from other virtual objects because of their greater emotional and social connection points, which more closely resemble real human users, with movements that mimic human behavior [43]. Given their potential impact on time perception, it is essential to investigate how these surrounding avatars affect participants in VR gyms. This study addresses this gap by examining two key attributes of the surrounding avatars: motion speed and exercise intensity. We aim to provide insights that could guide the design of more engaging and effective exercise experiences in future VR gymnasiums.

## Methods

To better understand the effect of surrounding avatars on time perception in VR gyms, we focused on how the motion speed and exercise intensity of the surrounding avatars influenced the participants’ time perception. Additionally, we focused on how the participants’ states affect these effects. The entire experiment was conducted using a VR gymnasium. The speed of motion of the surrounding avatars and the weight of the barbell changed within the scenario. The cycling task was chosen for our study because it is a common task in gyms, may have lower interference in duration judgment, and is safe when wearing a head-mounted display (HMD). According to time psychology, some studies distinguish between two main types of time perception [44]. Therefore, we followed the time psychology method and focused on our hypothesis from two perspectives. Duration judgment refers to an individual’s objective estimation of the duration of a specific period. The second part concerns the feeling of time passage, which is an individual’s subjective perception of the rate at which time passes. We also focused on the participants’ state; therefore, the hypotheses were set to test whether cycling on the bike provided a more immersive atmosphere in the VR gym to make participants more focused on the VR gym and the surrounding avatars exercising. Simultaneously, according to the internal clock mode theory, it can be explained that the sports state might increase the participants’ heart rate, and further, their higher accumulation of pulses will increase their time perception [45]. Thus, we formed the following hypotheses:

- H1: The fast-motion speed condition of the surrounding avatar leads to participants making longer duration judgments (H1-1) and feeling time passage faster(H1-2).
- H2: The high-intensity exercise condition of the surrounding avatar leads to participants making longer duration judgments(H2-1) and feeling time passage faster(H2-2).
- H3: The cycling state will increase the effect of the surrounding avatar on the participants’ time perception compared to the sitting state.

### Software and hardware

**Fig 1:**
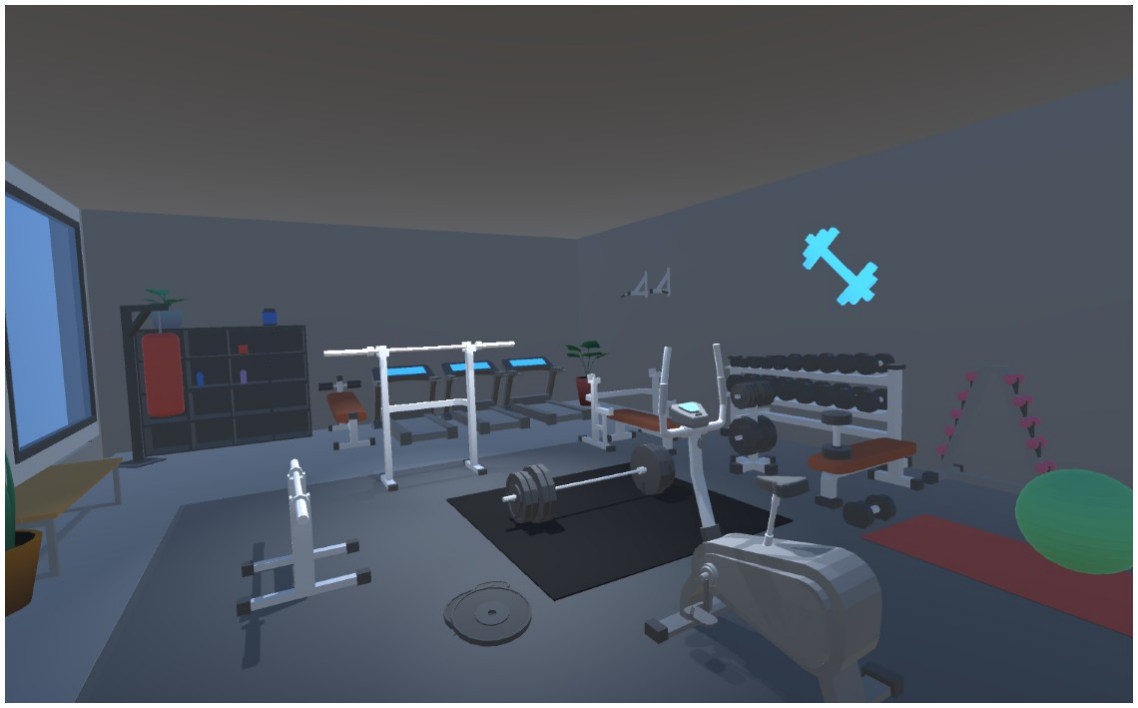
The VR gym design 3D model of training machine. These were used as the main scenario during the whole experiment.

A fitness bike (ALINCO, AFB6119) with a magnetic load system was used by the participants for cycling. The maximum load capacity of this fitness bike was 120 kg, and various sports modes could be selected. The entire experimental system ran smoothly on a desktop PC(Intel(R)Core^™^ i7-12700, 16 GB RAM, NVIDIA GeForce RTX 3060). We chose META QUEST 2 as our Head mount display device to provide participants with a truly immersive virtual experience. This HMD has a 90° field of view, a resolution of 1832 × 1920 pixels per eye, and a 90Hz refresh rate. To creating the experimental environment, we have chosen Blender (v. 3.6.5.0) as the main tool to create a the 3D model and Unity3D (v. 2021.3.9f1) as the main development tool. Using 3D modeling, we constructed a VR gymnasium environment consisting of many different training devices, such as treadmills, barbells, and chest push frames. Regarding the design of the entire virtual space, we opted for a low-poly aesthetic to craft 3D models constituting the VR gym. While this design choice lent a distinctive style to our environment, it could potentially trade off the degree of realism. However, based on our subjective experience, this stylistic decision did not markedly affect the overall virtual immersive. Unity3D was used to create an entire environment and arrange each position of the training device and VR application (Fig 1). A specific experimental room was laid out to allow participants to easily slip into the VR gymnasium. A virtual bicycle station was placed within the experimental area, permitting participants to engage in experimental tasks while immersing themselves in the VR gymnasium scenario.

**Fig 2:**
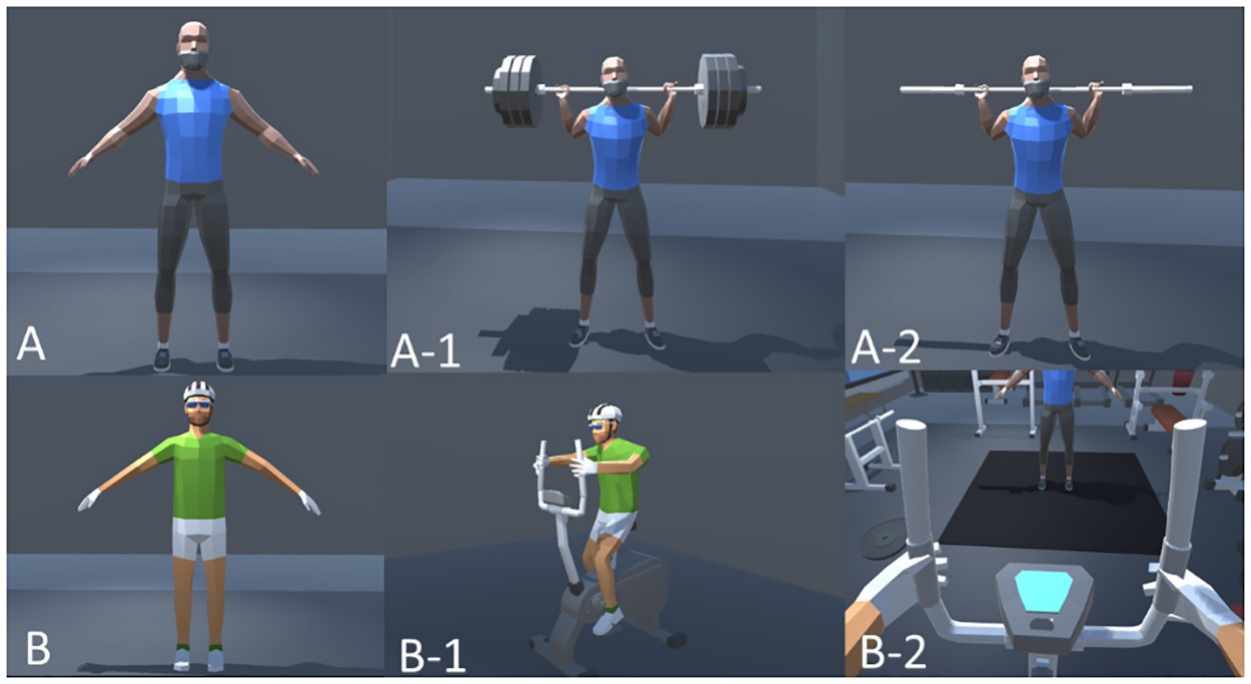
The Avatar used in the experiment: A) Surrounding avatar A-1) Surrounding avatar under heavy-workload condition A-2) Surrounding avatar under light-workload condition B) Participant’s avatar B-1) Participant’s avatar with cycling gesture B-2)User’s viewpoint.

For the surrounding avatars and the avatar representing participants, both of them used low-poly style models from “Low Poly Animated People” [46], which can fully improve visual coherence and presence in VR gym environments. In addition, the outside appearance of the surrounding avatar assumed that of an athlete, which was more suitable for VR gym (Fig 2A and 2B). The default surrounding avatar was positioned centrally in the VR gym to ensure maximum visibility. Two barbells with different numbers of weight pieces be used to do squat by the surrounding avatar: one with three big plates and another smaller plate on each side of the barbell pole, another one only has barbell pole-like (Fig 2A-1 and 2A-2). The surrounding avatar animation, a barbell squat, was obtained from the Mixamo website [47].

To represent the participants within the virtual gym, we crafted an avatar situated on a stationary bike (Fig 2B-1). The participants perceived the VR gym through a first-person perspective to observe the environment, allowing them to change their sight view by simply moving their heads (Fig 2B-2). Considering the nature of the experiment, whether it was a sitting or cycling task, the participants remained postured on a stationary bike until each experimental section finished. Consequently, the participant’s avatar was fixed to the virtual station bike in the VEs (Fig 2B-1).

### Participants

We recruited 28 participants for the experiment through an online communication group. The recruitment period ran from September 14 to October 5, 2023, and the experiment was conducted from September 23 to October 10. The average patient age was 26 years, with a standard deviation of 2.4 years. Among the participants, there were 9 females and 19 were male. All the participants were unaware of the purpose of the study. The participants had normal or corrected-to-normal vision. All participants provided written informed consent. Participants were free to withdraw at any time during the experiment. This experiment was approved by the Research Ethics Committee of the Japan Advanced Institute of Science and Technology and was conducted in strict compliance with its contents.

### Experimental conditions

In real-world gyms, people typically do squats or other resistance exercises following a mode like one training set with one rest set, which is called “Rest between sets (RBS)” [48]. For squats, people typically spent 20, 30, and 40 s to complete each training set.

We conducted a within-participants experiment using three independent variables. The first was motion speed with three levels: I) slow motion speed, II) medium motion speed, and III) fast motion speed. The frequency was used to define three levels: for slow motion speed, the surrounding avatar performed 19 squats per minute; for medium motion speed, it was performed 28 times per minute; and for fast motion speed, it was performed 38 times per minute. The motion speed of the surrounding avatar was based on “The Ruffier Squat Test”, which is a quick fitness test that checks heart health by measuring heart rate before and after doing squats. it needs people to squat 30 times in 45 seconds. However, since this is a high-intense test, we reduced the frequency to 38 times per minute as the fast-motion speed condition in our study. Furthermore, a 30% reduction was applied to create the other two conditions [49]. The second independent variable was the exercise intensity of the surrounding avatar, with two different exercise intensities: I) low and II) high. For the low exercise intensity, the surrounding avatar lifted a barbell pole without any extra weight plates; for the heavy intensity, the surrounding avatar lifted a barbell pole with three big and two small weight plates on each side of the barbell. The last independent variable was the state condition of the participant. The participants observed and estimated the duration while either sitting on a stationary bike or cycling on a stationary bike.

In designing the duration of each trial, we took into account previous research by Landeck et al [36]. The duration of each trial was set to 20, 30, and 40 seconds. Each default time that we set for each trial was presented with equal frequency and in a random sequence. So for each experiment session, participants observed the surrounding avatar doing exercise with RBS, and the task was to estimate time for each exercise set, with the rest time allocated for answering questions. This task design makes the duration judgment of each workout set more relevant to actual gymnasium training styles. Additionally, previous research found that judgment long term is more affected by emotions [50]. Relatively, short-term duration judgment can reflect people’s time perception more accurately. Meanwhile, Droit-volet et al. found that the feeling of time passage had a positive correlation between several seconds and several minutes of judgment [50, 51]. Also, when time exceeds 1 scend duration judgment and feeling of time passage also correlate.

### Procedure

**Fig 3:**
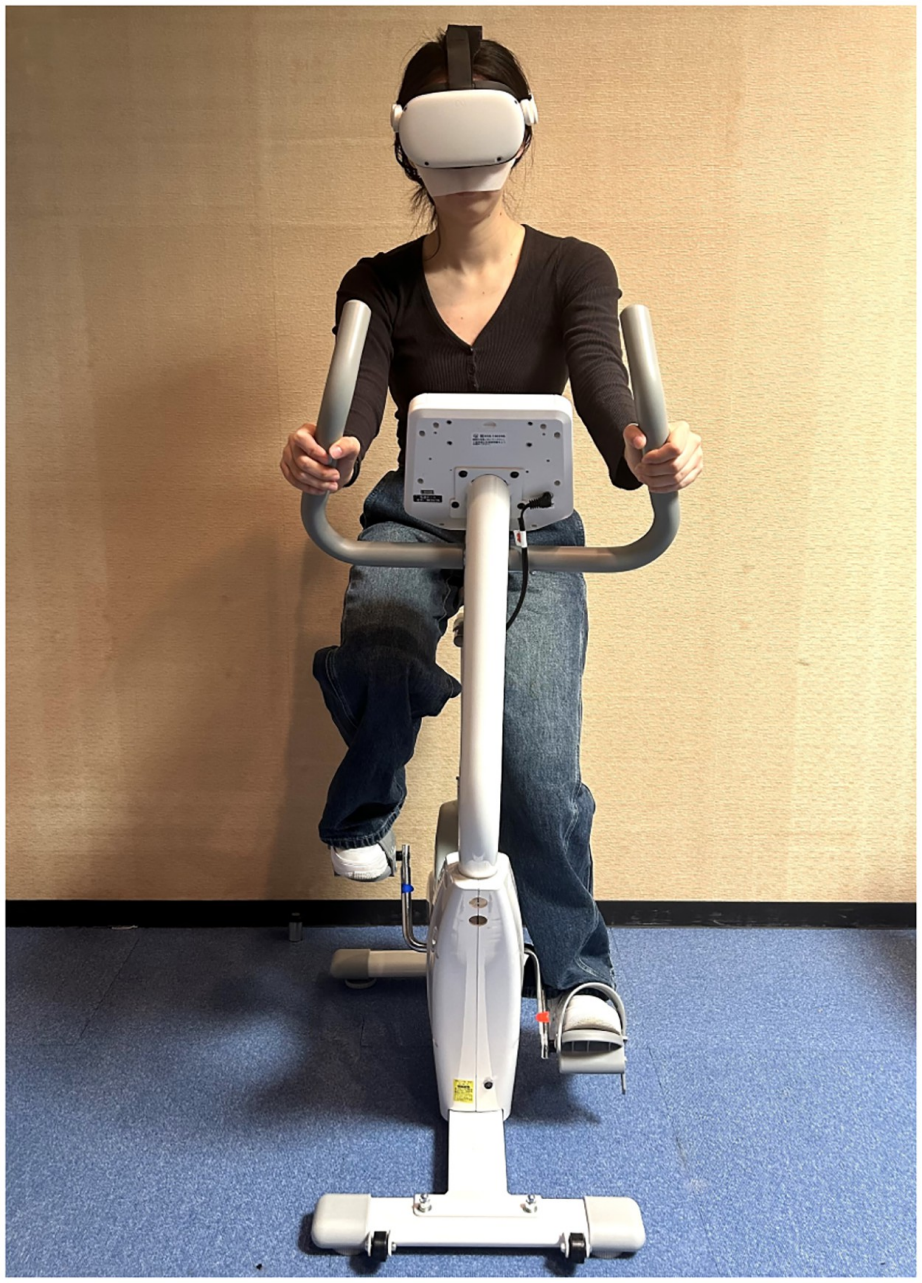
The procedure for the VR experiment.

**Fig 4:**
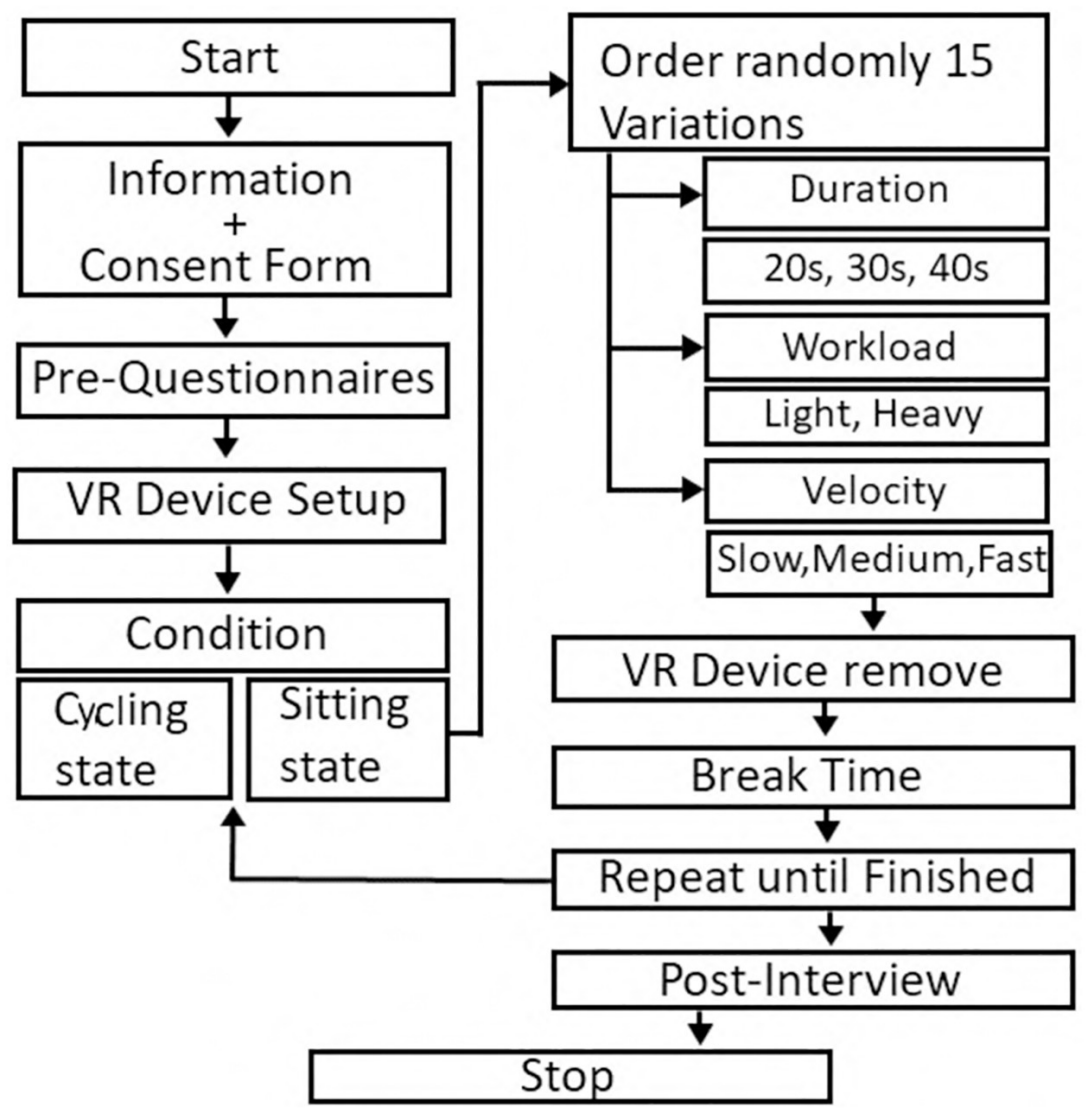
The scene of the experiment.

The entire procedure of the experiment is shown in Fig 3. Participants first signed a consent form after the experiment explanation after they completed a demographic questionnaire to provide basic information. Next, they then wore disposable socks and indoor sneakers, adjusted the bike seat height to match their height, and put on the HMD (Fig 4).

First, they had a practice session to get used to the task, which included 6 trials and answering questions 6 times. Following the practice session, participants took a 5-minute rest in a separate room. The main experiment consisted of four sessions: two cycling sessions and sitting sessions. To reduce the potential bias caused by cycling fatigue, there were no two consecutive cycling sessions. For the sitting session which lasted 18 minutes, participants sat on the bike and were first shown a hint scene: “You are in the observation stage, please observe and familiarize yourself with the environment,” to help them immerse into the VR gym. After that, participants entered and observed the environment for 1 minute and familiarized themselves with the VR gym. Then, they began the first trial. After each trial, they answered two questions. After answering, they proceeded to the next trial. This process continued until all 19 trials were completed, one of the trials was without avatar condition as the baseline in the experiment. The cycling sessions, lasting around 19 minutes, differed only in the hint scene: “You are in the warm-up stage, please start cycling. The experiment will start after 2 minutes warm up.” The remaining trials followed the same format as the sitting sessions. The stationary bike was set to a 30-watt mode to ensure all participants consumed the same amount of energy regardless of their pedaling frequency.

After each session, the experimenter helped participants remove the HMD. There was a 10-minute rest period between sessions, during rest time, participants wore eye masks and headphones to listen to light music, promoting deep rest and reducing fatigue and outside interference. Throughout the experiment, participants completed an equal number of trials involving combinations of three motion speeds, two exercise intensities, three observation times, and one blank scene without any surrounding avatar, counterbalanced in randomized sequences, totaling 76 trials. After the experiment, a brief interview was conducted, which focused on the effect of the surrounding avatar’s motion speed and exercise intensity on their perception of time.

### Measures

In this experiment, we designed the following two questions to measure how long the participants felt in each trial and their perception of time passage, as used by Lugrin et al., Unruh et al., and Landeck et al. [20, 22, 36, 37]. Both of these questions were displayed in Chinese and were only explanation during the experiment. All participants fully understood the meaning of the two questions by reading the experiment explanation document.

- Q1: Intuitively (no more thinking), how long do you think you existed in the VR gym? Please use seconds as the unit of estimated time and say it directly and orally.
- Q2: From your physical feeling, how fast is the time passage?

Q2 is a 7-point Likert scale adopted from the Inventory on Subjective Time Self and Space (STSS) [52]. Q1 was used to measure participants’ subjective time perception, “individual’s objective estimation of the duration of a specific period”. Q2 was used to measure “participant’s subjective perception of the rate at time passage”. Based on the specific situation in this experiment, we have revised the question slightly. For Q1, we added a description of how to answer the question orally and the details of duration judgment from existing in the VR gymnasium. For Q2, “from your physical feelings” was added; participants needed answers from their physiological responses rather than relying on their brain’s thinking to get more accurate and avoid errors on the feeling of time passage.

#### Statistical analysis

To evaluate the effects of the conditions on the percentage duration judgment, we applied three within-subject variables: motion speed, intensity, duration, and participant state. We conducted a repeated-measures analysis of variance (ANOVA) to investigate the effects and interactions of these independent variables on the dependent variables, including time estimation and feeling of time passage. Normality was then tested using the Shapiro-Wilk Test. Sphericity was assessed using Mauchly’s test and appropriate corrections were applied when violations were detected. All analyses were conducted using the R programming language in RStudio as the development environment.

## Results

### Duration judgment

**Fig 5:**
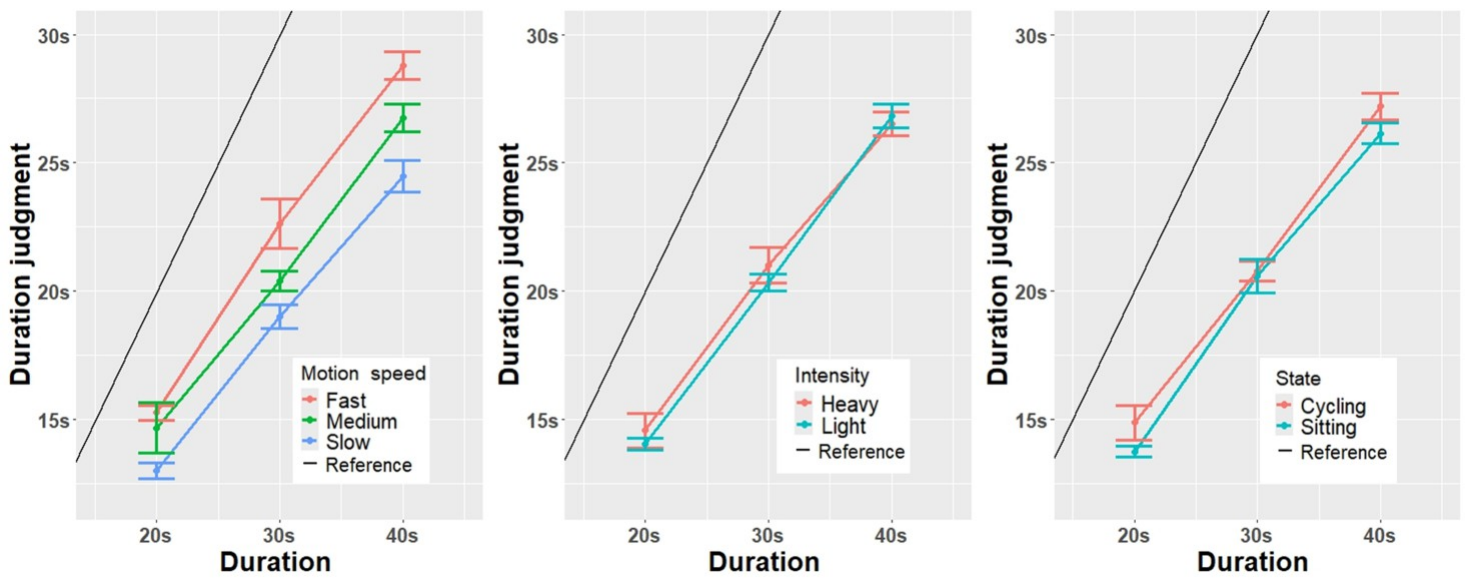
Main results of the dependent variable duration judgment: Scores on motion speed, exercise intensity, and participants’ state, with error bars.

The results of the duration judgment are presented in Table 1 and Fig 5. Although the assumption of normality was violated for the dependent variable (duration judgment), we converted the data logarithmically [53]. After conversion, most data were normal. However, several conditions were still violated; therefore, we also proceeded with ANOVA because it is quite robust in terms of normality [54, 55].

**Table 1:** The result of duration judgment.

	F-value	Degrees of Freedom (df)	*P*-value	Effect size($\eta_{p}^{2}$)
Motion speed	29.98	35.98	.001	0.051
Exercise intensity	0.03	27	.865	0.001
Participant’s state	0.52	27	.479	0.029
Duration	1362.87	46.35	.001	0.407

the results of different surrounding avatar conditions on the comparison of duration judgments.

For the comparison between the surrounding avatar’s different motion speeds, there was a significant effect on the duration judgment (F(1.33, 35.98) = 29.98, p < .001, $\eta_{p}^{2}=0.051$). After that, there also was an interaction significant effects between motion speed and participants’ state (F(3.51, 84.92) = 8.61, *p* = .005, $\eta_{p}^{2}=0.005$). Furthermore, there was a significant difference in duration (F (1.72, 46.35) = 1362.87, *p* < .001, $\eta_{p}^{2}=0.407$) on duration judgment.

For the comparison between the surrounding avatar’s exercise intensities, the results revealed no significant difference in the duration judgment between exercise intensities (F(1, 27) = 0.03, *p* = .865, $\eta_{p}^{2}=0.001$). For the comparison of participants’ states, the result presented that there was no significant difference (F(1, 27) = 0.52, *p* = .479, $\eta_{p}^{2}=0.001$).

The Bonferroni test was used to perform post hoc tests following the significant main effect of motion speed on duration judgment. Bonferroni-adjusted post hoc comparisons revealed that all pairwise differences between the surrounding avatar motion speeds for three durations were significant. The analysis revealed distinct differences among the various motion speed conditions. the fast motion speed condition (M = 3.02, SE = 0.04) compared with the medium motion speed condition (M = 2.94, SE = 0.04) (fast motion speed >medium motion speed (t(27) = 5.112, *p* < .001)), the medium motion speed condition (M = 2.94, SE = 0.04) compared with the slow motion speed condition (M = 2.84, SE = 0.05) (medium speed >slow speed (t(27) = 4.558, *p* < .001)).

To further understand the interaction effects on the participants’ states and the surrounding avatar’s different motion speeds, we also performed a post hoc comparison with Bonferroni adjustment. The results showed that at the slow motion speed, the cycling state (M = 2.88, SD = 0.45) was not significantly different(t(27) = -1.993, *p* = .0565) from the sitting state (M = 2.80, SD = 0.42). Similarly, under the medium motion speed, the sitting condition (M = 2.93, SD = 0.38) compared with the cycling condition (M = 2.95, SD = 0.43) was not significantly different (t(27) = -0.632, *p* = .532). In addition, under fast motion speed, the sitting condition (M = 3.03, SD = 0.371) was significantly higher (t(27) = 0.971, *p* = 0.340) than the cycling state (M = 3.00, SD = 0.42).

#### Compare with actual duration

From Fig 5, we found that almost all conditions of duration judgment were underestimated. Thus, we performed a one-sample t-test under all conditions to determine whether the duration judgment and actual duration were statistically significant. The results showed a highly significant difference, with a mean difference of 9.44 (t(2016) = 43.521, *p* < .001). This indicates that duration judgment was significantly lower than the actual duration for all experimental conditions.

Except for the conditions of the surrounding avatar’s motion speed and intensity, we conducted another trial in each experimental session without any surrounding avatar, as the reference condition. We also conducted a one-sample t-test to evaluate whether the duration judgment and actual duration were significantly different in the reference condition. The results revealed a significant difference between duration judgment and actual duration, with a mean difference of 6.875 (t(111) = 12.808, *p* < .001), and the duration judgment was significantly lower than the actual duration.

#### Post hoc power analysis on duration judgment

To ensure that our sample size was adequate for detecting the expected effects, we conducted a post-hoc power analysis using statistical power and sample size calculation tools. Based on an effect size of 0.051, a significance level of 0.05, and a total sample size of 28 participants, the analysis yielded an actual power of 0.653 (65.3%).

### Feeling of time passage

**Fig 6:**
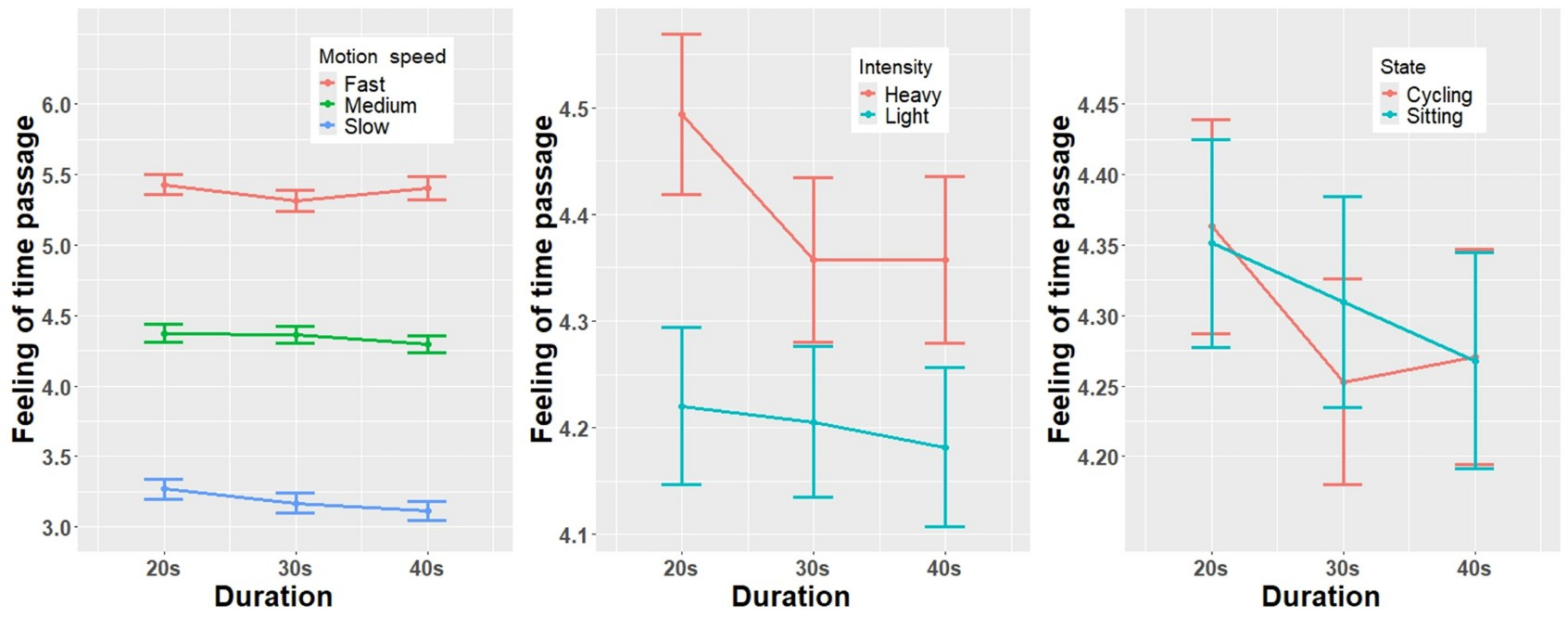
Main results of the dependent variable feeling of time passage: Scores on motion speed, exercise intensity, and participants’ state, with error bars.

The results of the feeling of time passage are presented in Table 2 and Fig 6. For the dependent variable of the feeling of time passage, the result of the assumption showed that normality was violated. Although the normality assumption was violated, we continued with ANOVA because of its robustness [54, 55].

**Table 2:** The result of the feeling of time passage.

	F-value	Degrees of Freedom (df)	*P*-value	Effect size($\eta_{p}^{2}$)
Motion speed	116.99	30.06	.001	0.437
Excercise intensity	6.25	27	.019	0.010
Participant’s state	0.02	27	.891	0.001
Duration	1.82	27	.177	0.001

the results of different surrounding avatar conditions on the feeling of time passage.

The results showed that the feeling of time passage was significantly affected by two independent variables related to the surrounding avatar, the motion speed (F(1, 27) = 116.99, *p* = .019, $\eta_{p}^{2}=0.01$), and the exercise intensity (F(1, 27) = 6.25, **p** = .019, $\eta_{p}^{2}=0.473$.

To explore the effects of the surrounding avatar’s different exercise intensities and speeds on the feeling of time passage, we conducted a post hoc pairwise comparison with Bonferroni adjustment, and the results showed that faster motion speed led to a higher feeling of time passage (fast motion speed>medium motion speed (t(1, 27) = -8.432, *p* < .001); medium motion speed>slow motion speed (t(1, 27) = -12.817, *p* < .001)).

For the participants’ states, there was no significant difference in the feeling of the time passage (F(1, 27) = 1.82, p = 0.177, $\eta_{p}^{2}=0.001$). There was a significant interaction between motion speed and participants’ state (F(1.78, 47.93) = 10.26, *p* < .001, $\eta_{p}^{2}=0.008$).). Other interactions did not show any significant differences. Subsequently, we performed a post hoc Bonferroni correction for the interaction effect of the participant’s state and motion speed. The results showed that under the slow-motion speed condition, the sitting state (M = 3.06, SD = 0.99) was significantly lower (t(27) = -2.305, p = 0.029) than the cycling state (M = 3.30, SD = 1.11). However, under the medium motion speed condition, the sitting state (M = 4.38, SD = 0.80) was significantly lower (t(27) = 0.656, *p* = 0.518) than the cycling state (M = 4.30, SD = 1.02). Under the fast-motion speed condition, the sitting state (M = 5.48, SD = 1.05) was significantly lower (t(27) = 1.683, *p* = 0.104) than the cycling state (M = 5.28, SD = 1.20).

#### Post hoc power analysis on the feeling of time passage

To ensure that our sample size was adequate for detecting the expected effects, we conducted a post-hoc power analysis using statistical power and sample size calculation tools. Based on an effect size of 0.473, a significance level of 0.05, and a total sample size of 28 participants, the analysis yielded an actual power of 0.997 (99.7%). This power exceeded the typical 80% standard, indicating that our sample size provided a high level of statistical power for detecting significant effects.

## Discussions

### Summary of experiment results

Our results strongly indicate that the attributes of the surrounding avatar significantly influenced the participants’ time perception. For the hypothesis that the participants judge the duration longer in fast motion speed and high exercise intensity conditions compared to slow motion speed and low exercise intensity conditions, responses to Q1 (“How long do you think you existed in the VR gym?”) showed significant differences between conditions with varying motion speeds, and the participants judged the duration to be longer in faster motion speed conditions. Thus, this result supports *H1-1*. However, the results did not show significant differences in the estimated duration between the two exercise intensity conditions; therefore, *H2-1* was rejected.

For the feeling of the passage of time, we hypothesized significant differences between the motion speed and exercise intensity conditions. Responses to “From your physical feeling, how fast is the time passage?” demonstrate significant differences between the different motion speed conditions. Participants felt time passed was faster in the fast-motion speed condition than in the slow-motion speed condition. Therefore, *H1-2* were supported. Meanwhile, for the exercise intensity conditions, high exercise intensity conditions led to a faster feeling of the time passage. Therefore, *H2-2* is supported.

For *H3*, we hypothesized that participant’s cycling states would lead to higher duration judgments and faster feeling of time passage. However, our results did not align with our expectations, as the findings indicated no difference in time perception between the cycling and sitting states. One possible reason that in this study the surrounding avatar attracts almost all the attention of the participants is that participants can not spare their attention to cycling or not cycling, so the participant’s state, cycling or sitting, can not affect people’s time perception.

### Difference between duration judgment and feeling of time passage

In this section, we discuss the effects of surrounding avatars on time perception. Our results showed that motion speed and exercise intensity played important roles in the participants’ time perception. However, the two attributes had different effects: while the motion speed affected both the duration judgment and the feeling of the passage of time, the exercise intensity only affected the latter. This difference may be due to the different mechanisms between duration judgment and the feeling of passage of time. Droit–Volet et al. examined the relationship between duration judgment and the passage of time with concurrent measurements and duration judgment and found that the feeling of time passage should be considered distinct. Moreover, duration judgment and the feeling of the passage of time lack a direct connection [56]. Another study has also highlighted that duration judgment is affected by the internal clock, and when the pacemaker of the internal clock increases, the emitting pulses speed up, and the duration judgment will be longer. However, the passage of time is affected by emotional valence, arousal, attention to time, and time expectation [57]. As evidence, patients with depression experience a slowing down of time passage, although they do not exhibit any deficit in duration judgment compared to healthy individuals [58].

Based on these ideas, in the next two sections, we separately discuss the effects of the attributes of the surrounding avatars on the two dependent variables.

#### Duration judgment

Our experimental results demonstrated that the speed of motion of the surrounding avatars significantly affected duration judgment, whereas exercise intensity had no significant impact on duration judgment. One possible explanation is that duration judgments are often affected by the temporal attributes of stimuli and factors that connect to temporal attributes, such as speed and frequency, but not by the magnitude of the stimuli. In Brown’s research, participants observed various stimuli displayed on a monitor, and the speed and number of stimuli were manipulated. The results indicate that the former affected duration judgment, whereas the latter did not affect the duration judgment [59].

Our results show a similar phenomenon. Regarding the speed of the surrounding avatars, faster-surrounding avatars made the visual stimuli move faster, which further resulted in a higher duration judgment. In addition to Brown’s research, which was conducted using 2D monitors, the research conducted by Landeck et al. also supports our ideas. They demonstrated that altering the speed of a virtual tunnel shuttle influenced duration judgment [36].

For exercise intensity, while the high exercise intensity condition provided a high exercise intensity from visual stimuli because of the larger size of the barbell, it did not enhance the temporal attributes of stimuli, such as motion speed or frequency, throughout each trial. Therefore, exercise intensity did not significantly affect the participants’ duration judgment. Similar research can be found in a study conducted by Unruh et al., which provided varying levels of avatar embodiment and did not alter duration judgment [22].

#### Feeling of time passage

Our experimental results indicated that the surrounding avatar’s motion speed and exercise intensity affected participants’ feelings of time passage. The comparison of temporal expectations is crucial in the feelings of the passage of time. This refers to the psychological phenomenon in which individuals anticipate or expect time consumption based on situational cues or prior experiences [60]. When people perceive that their subjective feelings of duration exceed their expected or anticipated duration, they often experience a sense of time dilation and perceive time as passing more slowly. Conversely, when the actual time falls short of the anticipated duration, individuals may feel that time passes more quickly, resulting in a sense of time compression. Discrepancies between anticipated and actual time duration represent pivotal determinants that influence individuals’ feeling of time passage [61].

In this study, it is likely that participants used squats as a reference to judge time. Since squats were the only available stimulus, participants had a sense of how fast of time passage was.

For the motion speed of the surrounding avatars, a fast-moving avatar shortens the duration of each squat. Therefore, for a fixed number of squats, the participants observed a shorter duration compared to the expected consumed time of those squats. This may explain why the participants perceived the passage of time to be faster.

However, for the exercise intensity of the surrounding avatar, the expected time consumed for a one-time squat depended on the weight of the barbell. The expected consumption time for heavyweight materials may be considered greater than that for lightweight materials. At the same motion speed, the participants observed the same duration for both exercise intensity conditions. However, the expected durations of the two exercise intensity conditions were different; therefore, the comparison between the observed and expected duration may be different. Consequently, the feeling of time passage was different, while the feeling of time passage in the high-intensity exercise group was higher than that in the low-intensity exercise group.

### Relationship between participant’s state and motion speed

Our findings revealed a significant interaction effect between the participants’ states (sitting or cycling) and the motion speed of the surrounding avatar. This interaction affects duration judgment and the subjective feeling of the passage of time; thus, we supposed that it could be shared by the same underlying factor.

Specifically, the avatar’s motion speed and the participants’ state jointly influenced the feeling of time passage and duration judgment. This could be due to the different motion speeds of the surrounding avatars, which attracted the participant’s attention to the VR gymnasium. For instance, as explained in the previous section, factors related to temporal clues affect people’s judgments of duration and their feelings about the passage of time. Therefore, in the condition of fast motion speed, the surrounding avatar’s squatting speed attracts the participant’s attention because there are many temporal clues released by the avatar’s fast motion speed. Thus, whether the participants stated cycling or sitting alone did not affect their time perception. The converse holds in the slower motion condition; the slower motion of the avatar provides less temporal stimulation, allowing participants to be affected by the surrounding avatar and cycling. During cycling, the brain processes more information, including temporal aspects, such as cycling frequency and body rhythm, which may contribute to a heightened perception of time. Previous studies have shown that physical activity can influence the judgment of duration. In contrast, when sitting, the lack of additional stimuli beyond the avatar may result in less pronounced effects on time perception [4, 26].

### Compare with actual duration

Our results indicated that all experimental conditions on duration judgment were underestimated, even without the influence of surrounding avatars, participants consistently underestimated the duration in the reference conditions. This finding aligns with previous research on time perception in VR, which can distort an individual’s perception of time, independent of other factors [18]. In VR, people often feel that time passes more quickly because they lose access to natural time cues, such as bodily rhythms, and environmental signals, such as sunlight or other factors. This phenomenon has been observed in similar contexts; for example, Lugrin et al. found that people waiting in VR perceived time as shorter than waiting in the real world under identical conditions [20]. This also explains why nearly all the conditions for duration judgment were underestimated.

### Contribution to VR design

Our findings suggest that the surrounding avatars in a VR gymnasium may strongly affect people’s time perception. These findings may contribute to the VR design in two ways.

#### VR gym

As mentioned above, painful sensations and time distortion due to vigorous exercise may indirectly affect willingness to continue exercising. Our study provides a possible solution to the problem of time distortion in VR gyms. For future VR gymnasium scenario designs, the designer may based on the user’s perspective to adjust the parameters of the surrounding avatar’s motion speed and intensity in the VR gym. when users need to adjust their time perception, our study provides some scheme or possibility to change people’s time perception. To adjust the surrounding avatar in the VR gymnasium to help them relieve bad experiences from time distortion induced by exercise fatigue and to extend their willingness to complete their exercise goals. However, while this is a statistically valid method to adjust people’s perceptions of time, it doesn’t rule out individual differences.

#### A new zeitgeber

Another contribution is that our results indicated that surrounding avatars can be seen as a “zeitgeber.” A zeitgeber is a thing that contains time cues and can synchronize people’s inner clocks and regulate their sense of time. Previous research has shown that virtual objects with explicit time clues, such as clocks and virtual objects performing pendulum motion, can be seen as the “zeitgebers” [37]. In addition to non-living things, our findings showed that the participants used surrounding avatars, whose movements were not consistently static, to judge the duration. Thus, the surrounding avatar can be recognized as a zeitgeber in VR.

### Limitation and future works

In this study, we gained detailed insights into time perception in VR gyms. We believe that our research results represent a first step toward helping realize the effectiveness of the surrounding avatar while working in VR gyms. However, this requires a long time. This section highlights some of the limitations of this study.

First, the surrounding avatar played the role of a zeitgeber in the VR gyms, affecting participants’ time perception. In this study, squats were selected as the exercise for the surrounding avatar. Compared with squats, which have a regular cycle, other exercises such as running on a treadmill [62], do not have a regular cycle. Thus, it is necessary to investigate whether the attributes of the surrounding avatars in these cases have similar effects in the future.

Second, the effects of motion speed and exercise intensity on time perception may not be linear. We selected a common range of motion, speed, and exercise intensity as the conditions. However, it is necessary to further investigate whether these effects persist under extreme settings. It is possible that when the surrounding avatar’s motion speed and exercise intensity are significantly higher than usual, the effects may diminish or disappear because the participant may not consider the surrounding avatar zeitgebers in such an unusual situation. Therefore, in future work, we will replicate the experiment in a wider setting to explore these effects.

Third, the surrounding avatar was always in a fixed position throughout the trials. This is somewhat artificial compared to a real VR gym situation. A real VR gym might contain many interactions between users and surrounding avatars. Such social behavior might be crucial in time perception because it induces participants to have a better immersive feeling. Additionally, previous studies have shown that time perception is generally associated with the environment and situations [63]. Hence, further research in more interactive settings is required.

Fourth, for the experimental design of each trial, we followed Landeck et al. ’s design in the 20s, 30s, and 40s [36], and used this trial design to simulate the way how the people work on anaerobic exercise in a real gym. However, it was relatively short to measure people’s time perception about the content of exercise. The purpose was to obtain precise time perception and simulate more real situations in a VR gym. However, further experimentation and long-time judgment are required to test the same conditions in a more realistic time condition.

## Conclusion

This study explored the effects of the surrounding avatar’s motion speed and exercise intensity on time perception in a VR gym. We demonstrated that the motion speed of the surrounding avatars significantly influenced both the judgment of duration and the feeling of the passage of time. Faster motion speeds led to longer judgments and a faster perceived time passage. However, exercise intensity only influenced the feeling of time passage, with higher intensity leading to a faster perceived time passage, but not affecting duration judgment.

Additionally, our findings suggest that the interaction between the participants’ states (cycling or sitting) and the motion speed of the surrounding avatars can influence time perception. In particular, the effect of motion speed on time perception was more pronounced when participants were cycling, likely because of the additional temporal stimuli provided by the physical activity.

Our research offers valuable insights for designing future VR gyms, suggesting that adjusting the parameters of the surrounding avatars can help alleviate negative experiences of time distortion and enhance users’ willingness to continue exercising. Furthermore, this study introduces the concept of surrounding avatars as potential “zeitgebers” in VR environments, offering a new perspective on how time cues can be integrated into virtual settings to regulate the users’ sense of time.
